# Nursing strategies for child health surveillance^1^

**DOI:** 10.1590/1518-8345.2434.3007

**Published:** 2018-07-16

**Authors:** Marina Sayuri Yakuwa, Sarah Neill, Débora Falleiros de Mello

**Affiliations:** 2 Doctoral student, Escola de Enfermagem de Ribeirão Preto, Universidade de São Paulo, PAHO/WHO Collaborating Centre for Nursing Research Development, Ribeirão Preto, SP, Brazil. Scholarship holder at Conselho Nacional de Desenvolvimento Científico e Tecnológico (CNPq), Brazil.; 3 PhD, Associate Professor, Faculty of Health and Society, University of Northampton, Northampton, England.; 4 PhD, Associate Professor, Escola de Enfermagem de Ribeirão Preto, PAHO/WHO Collaborating Centre for Nursing Research Development, Ribeirão Preto, SP, Brazil.

**Keywords:** Child, Child Care, Surveillance, Nursing, Primary Health Care, Comprehensive Health Care

## Abstract

**Objective:**

to appreciate the strategies promoted by nurses in the context of child health surveillance relevant to early childhood development.

**Method:**

this is a qualitative study with an inductive thematic analysis of the data, based on the conceptual principles of child health surveillance, and developed through semi-structured interviews with Brazilian nurses working with families in primary health care.

**Results:**

the nurses’ strategies in favor of child health surveillance focus on actions that anticipate harm with continuous follow-up and monitoring of health indicators. The process of child growth and development is the basis for responses and benefits to health, connection with the daily lives of families, active search, articulations between professionals and services, access to comprehensive care, and intrinsic actions between promotion, prevention and health follow-up.

**Conclusion:**

child health surveillance actions developed by nurses with families involve knowledge sharing, favor the resolution of problems, increase child health indicators, and strengthen the relationship between health and children’s rights, which support the promotion of development in early childhood.

## Introduction

Health promotion, disease prevention and the early detection of physical abnormalities and developmental problems characterize child health surveillance programs^1^. Growing evidence has emphasized that timely and early intervention can change the lives of children, particularly the less favored ones^1^, and is essential to obtain a positive impact on human development from early childhood, from zero to six years of age^2^. Global initiatives have encouraged practices to stimulate early childhood development supported by multisectoral structures^2^ ^-^ ^3^, including health actions, nutrition, access to services, safe and affective environment, advocacy for child rights, protection and learning opportunities^4^.

Comprehensive health care for children aims to reduce childhood morbidity and mortality rates through the incorporation of new technologies, reorganization of the health system, and the involvement of various social agents and segments^5^. Estimates for 2030 indicate that under-five mortality is linked to prematurity, post-neonatal pneumonia, and intrapartum complications^6^. Global and local efforts to improve children’s health and development are therefore fundamental.

Primary Health Care (PHC), as coordinator in the organization of health systems, has assumed the important task of providing universal access and coverage of health services, with more solid and equitable health systems as a guiding framework for global development^7^. In the Brazilian reality, since 1994, the Family Health Strategy (FHS) has been implemented in order to strengthen the work with families, reorganize the health care model and propose significant changes in the context of primary health care, in line with the precepts of health surveillance^8^ and with a new paradigm focused on the humanizing and holistic practice^9^.

In the scope of primary health care and child health surveillance, nursing professionals have the responsibility of providing humanized care and support to children and their families, valuing the biopsychosocial well-being, identifying and intervening in needs and vulnerabilities^10^ ^-^ ^13^. In the present research, the understanding is that the nursing care for children’s health has been transformed by improvements in health and by the strengthening of shared knowledge in the context of working with families, such as it happens in the FHS, bringing subsidies for the reconstruction of health practices. Faced with the relevance of early childhood and health actions, nutrition, access to services, advocacy of child’s rights, and child protection^2^ ^-^ ^4^, it is critical to explore and detail the contributions of nursing practices to promoting development at this stage. 

Thus, this study had as objective to learn the care strategies developed by nurses in the context of child health surveillance, which are important for early childhood development.

## Method

This is a qualitative study with thematic analysis of the data, based on the conceptual principles of child health surveillance^1^ ^,^ ^8^, focusing on actions that anticipate damages or injuries and interventions for promotion, prevention and continuous monitoring in search for integral health care.

The research was developed in FHS units of a medium-sized Brazilian municipality where this programmatic guideline was implemented in 1999. The municipality has 14 health units of this nature, with a coverage of 17.3% in relation to the general population.

The following inclusion criteria were used to select the study participants: nurses who work in FHS units; nurses working with the FHS for at least six months; nurses who participate in child health care (from 0 to 12 years old); and nurses who voluntarily accepted the research invitation. The exclusion criteria were: nurses on sick leave or away from work.

The 14 FHS units in the municipality have 25 family health teams and a total of 25 nurses. The invitation to participate in the study was personally made by the first author to all the nurses of the Family Health Units (FHU) in that municipality, explaining the objectives of the research and giving them an Informed Consent Form. There were 3 nurses who refused to participate, and one was on sick leave during the data collection. After accepting to participate, and after signing the Informed Consent Form, the participants received a copy with the researcher’s signature. Thus, 21 nurses who met the inclusion criteria participated in the study. Data collection was terminated based on exhaustion, after approaching all eligible subjects.

Data collection was performed through semi-structured interviews, using the following questions: 1- In your opinion, what does child health surveillance in the family health strategy include? 2- What care strategies do you use for child health surveillance? Additional questions were asked to the nurses to clarify doubts and to deepen their professional experiences. The interviews were carried out from January to April 2014, in a private room in the work environment, and lasted from one to one and a half hours. Interviews were previously scheduled with the nurses, recorded in MP3 format, and transcribed verbatim. After transcription and analysis, they were deleted.

In this study, data were treated in a qualitative way, with the investigation of relevant aspects of the strategies used by the nurses to provide care for children, in the context of the FHS, based on the premises of health surveillance under PHC. In the analysis of the qualitative data, we used thematic inductive content analysis^14^. In the inductive model, the identified themes are extracted from the data, in which the inductive analysis represents a process of coding of the data, which are not fixed a priori, that is, the coding is directed and based on the data itself^14^. In this research, the elements of the practices of nurses were identified, analyzed and reported from the collected data, culminating in themes that translate significant parts and are based on the data set.

The present study was approved by the Research Ethics Committee (REC) of the University of São Paulo at Ribeirão Preto College of Nursing (REC nº 289/2013) with the use of an Informed Consent Term, guaranteeing the confidentiality of the data collected.

## Results

The characteristics of the 21 participating nurses who work in family health units are initially presented.

All participants were female and the age ranged from 27 to 56 years. The time ellapsed after graduation ranged from 5 to 32 years. Regarding the places of nursing training, 19 came from nursing schools in the local municipality of the study and two from other Brazilian cities. The time working at the FHS ranged from 8 months to 16 years. In relation to postgraduate training, specificifically focusing on children’s health and/or family health contents, 12 nurses reported the following specializations: Specialization in Public Health, Family Health, Management in Nursing and Psychiatric Nursing; Master’s degree in Family Health; PhD in Public Health, in addition to refresher courses. The other 09 nurses had attended refresher courses offered by health and education institutions.

**Figure 1:**
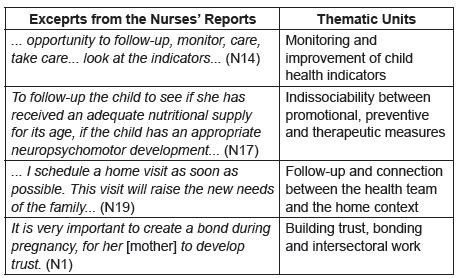
Presentation of excerpts from the reports of the interviewed nurses and their respective thematic units. Ribeirão Preto, SP, 2017

In the analysis of qualitative data, the significant dimensions were grouped into four thematic units, which were built from interviews reports, according to Figure 1.

The results were, therefore, grouped in the following thematic units: Monitoring and improvement of child health indicators; Indissociability between promotional, preventive and therapeutic measures; Follow-up and connection between the health team and the home context; Building trust, bonding and intersectoral work. These units portray strategies used by nurses who seek to transform child care based on health surveillance in the context of primary health care. Interviewees are identified with the letter N and a number (N1 to N21).

In the thematic unit Monitoring and improvement of child health indicators, it was highlighted that one of the strategies is the constant monitoring of children.

*To follow-up since when we start prenatal care. We evaluate all the possible complications, the possible diseases, the possible changes that may affect the baby and the woman. (N3); It is the opportunity to follow-up, monitor, provide care, look after, trying to look at both health indicators and social [indicators], and the whole issue of care. (N14).*

The emphasis on monitoring, to be attentive since prenatal care brings a vision of child health surveillance that encompasses the understanding of changes in health and social indicators over time and the need to anticipate and assess illnesses and complications. The nurses also pointed out that it is necessary to extend the professional gaze to surveillance, emphasizing that the nursing follow-up of children and their families is comprehensive.

*One can think of the context of extending the look towards care. It is to be able to look at the risks under which a child or any other person may be, and anticipate them. To see also what they [children] have as support, how is their health care, what they have to support them within that particular area that the child and the family are inserted... We have to be attentive to this: family planning, prenatal care, after childbirth, puerperium, child care and child growth and development. (N12); to follow up the child in the first phase of life, in order to be able to detect early problems. (N20); Surveillance is very broad, it involves not only to growth and development, but also psychosocial assessment. Thus, it’s very comprehensive. (N3); Surveillance is care... it is necessary to follow up and stay very close.. (N13).*

The health actions listed by the nurses reinforce their importance for the first years of the child’s life, for the early detection and needs of social support focusing on the vulnerabilities of children, reducing them and aiming at providing care.

In the thematic unit Indissociability between promotional, preventive and therapeutic measures, the strategy of interconnected attention emphasized a connection between the basic actions aimed at children’s health.

*Be aware of growth and development issues, whether the child is gaining weight adequately, whether she has no diarrhea, whether she has no vomiting, whether she can suckle well, whether the mother maintains exclusive breastfeeding... if the child has other siblings, if the mother is being able to provide that care, if she is being well cared for, if she has a supply of food, hygiene and sunbathing, everything that is recommended and is part of the conditions for a good development of the child. (N13); to follow up the child to see if he or she has adequate nutritional intake for the age, if he or she has the right neuropsychomotor development for the age, if she has conditions in the home and family to have a structure to grow and develop, if she lives in a healthy environment... cheking on factors that can interfere in the health of the child. (N17).*

The reports suggest that child health surveillance includes interrelated attention to childhood illnesses and environmental factors that interfere with the health of the child. There is also a focus on childcare consultations, which focus on the follow-up of child growth and development.

*Childcare consultations are an example of child health surveillance, to follow up, monitor the development, the growth of the child and social aspect as well. The social that surrounds the baby and the child throughout its development. So, it’s critical. I think the nurse is the center of it all. (N6); The child is constantly monitored in childcare consultations, if the mother is attentive to this, to keep the follow-up. (N9); to observe and guide on behaviors in relation to the best form of growth and development of the child... guidelines, information to make the child’s health reach the ideal. (N8).*

Childcare consultations are indicated as part of child health surveillance, highlighting and valuing the performance of nurses as fundamental agents of this process. Still with regard to promotional, preventive and therapeutic measures, a prominent strategy is the strengthening of unique plans of care.

*Our first role before a new mother, as soon as the baby is born, is to work with her... what are the tools she will acquire with us, or what she already has with her, in order to promote the health of the child. So, my role is to bring her to the care. (N21); each [care] plan is made according to the need of each family and child. Then, each child’s care project is drawn individually. (N15); generally, premature children have a different follow-up from the others; the consultations are conducted within shortest intervals. (N18).*

The reports provide a view of child health surveillance, which entails the elaboration of unique plans of care with the strengthening of parenting and the inherent tools to promote the health of the child and the family.

With regard to the thematic unit Follow-up and connection between the health team and the home context, the strategy highlighted was the realization of actions in home visits, active search and early identification of problems and illnesses at the home setting.

*I talk to them, and advise the community agent, I check if the mother is at home with the newborn and arrange a home visit as soon as possible. This visit will survey the new needs of this family and baby. And from that, we bring the information to the team at the moment we meet to discuss cases. (N19); We always do the active search for the missing ones, because we have a daily list. In the weekly meetings we always discuss cases of pregnant women and child care. (N1); one of necessary thing is each professional with his piece of understanding, working together, knowing what you are capable and joining the abilities, for the sake of the person. (N16); We usually use the active search strategy to avoid the risk that a child is left without any type of care. (N18).*

Actions at the home context are also seen as components of child health surveillance in the search for greater connection with the families’ daily lives and the guarantee of care and follow-up in situations of discontinuity of health care. In the articulation between the team and the home context, the discussion between professionals about the cases of families emphasizes the sharing of interprofessional knowledge and the practices of team work with a view to joint decision making.

In the thematic unit of Building trust, bonding and intersectoral work, the strategy to establish a bond between professionals and families were highlighted.

*It is very important to form a bond during pregnancy, for her [mother] to have confidence in you, the community agent in the area, so that she may feel free to ask questions, ask what she wants, to have openness. So you need to have a strong bond, that link. (N1); a follow-up from the beginning, special and continuous... and there is something good, patients come to the unit, they come back. There is a bond. (N10)*

The reports of the nurses reinforce the importance of positive interactions for child health surveillance, an aspect related to increased trust and freedom to share knowledge and doubts with families.

Another strategy highlighted was the articulation between services and health professionals and other social sectors.

*Communication between sectors, and among professionals; some sheets [record information] that we have created in the course of time, the interaction between nursing, doctors and community agents, the system [computerized health information system], all these aspects allow us to understand this process of children in the network. (N14); Health surveillance encompasses a bit of everything, epidemiological information, diseases of compulsory notification, it is this whole thing... it is fundamental to try to seek other services when necessary, in terms of referrals, or the social issue, to discuss with the social service staff, with the multiprofessional [interdisciplinary] team. (N5); for me, some epidemiological, sanitary and environmental surveillance comes into the scene. (N18).*

Nurses mention aspects of the organization and flow of information between health services and other sectors as part of child health surveillance, the importance of intersectoral work.

The results suggest that the child health surveillance developed by nurses who work with family health is characterized by constant monitoring, child care consultations, home visits, active search of children and pregnant women, articulation among health professionals and intersectoral actions, which seek to consolidate adequate responses to the health and development of children and their family.

## Discussion

In this study, the interviews with nurses working in FHS units made it possible to understand the professional strategies developed in favor of child health surveillance. The present research identifies strategies for the applicability of child health surveillance, focused on ensuring continuous follow-up, the anticipation of illnesses and complications, interrelated attention to essential actions of child health care, elaboration of unique plans of care, sharing of interprofessional knowledge and intersectoral actions. These aspects of different moments of attention to children’s health suggest primordial practices in the way of caring in the context of primary health care for children, in the pursuit of the increased benefits and consolidation of adequate responses to health and development in the early childhood, emphasized in this study.

Concerns about the health and development of children and prevention are present in other investigations^2^ ^-^ ^5^ ^,^ ^9^ ^,^ ^11^ ^-^ ^13^. Since the year 2000, the term ‘child health surveillance program’ has been expanded to ‘child health promotion program’^1^, with greater emphasis on early detection of harms and vulnerabilities. It is therefore necessary to ensure the survival of children, but also to offer the conditions to live with quality, to grow, to develop and to reach its full development potential^3^ ^,^ ^15^.

In this focus, the present study points out that nurses play a fundamental role among families, based on health surveillance, for resolute and individualized responses to the needs of early childhood, which is a noble period of human development. Thus, nursing actions in health surveillance interventions are relevant, considering the repercussions regarding the strengthening of good parenting practices, positive and affective interaction with children, reduction of stress and prevention of infantile injuries and violence, avoiding imbalance in the human development^10^ ^-^ ^13^.

A study^16^ pointed out the work of nurses in PHC incorporates promotion activities aimed at improving the social determinants of health, in situations of vulnerability, with advanced skills that include coordination, education, counseling, collaboration, connecting clients with services and advocacy. It further emphasized the importance of individual and community interventions resulting in increased access to care, reduced costs and salutogenic characteristics of empowerment for social changes^16^.

Childcare consultations were highlighted as part of the practice of child health surveillance, considering socioeconomic, environmental and cultural aspects, and provision of guidelines for mothers on breastfeeding, vaccination, hygiene, among others. The education of mothers/caregivers has been emphasized to give families subsidies for protective care to children’s health^2^ ^-^ ^4^ ^,^ ^10^ ^-^ ^11^. Home visits and the active home search were also emphasized in child health surveillance, in line with other international investigations that address these practices as relevant to evaluate the mother-child interaction and the attention to the child^17^, and to allow a closer understanding of health-disease determinants and contribute to the improvement of the trajectories of children, women and families^13^ ^,^ ^18^. 

This way of apprehending child health surveillance is in line with the scientific literature on this matter, advocated as a model of health care that has as its object the determinants of ways of life and health, living and working conditions, damages, risks and needs, as well as the active participation of citizens and of the health team^8^ ^-^ ^9^ ^,^ ^19^. In the present study, there are convergent results with what has been argumented on the nursing actions in child health care, with anticipated actions and prevention of intercurrences and complications of injuries, as an active component in the performance of surveillance^9^. A study^20^ showed that nurses who work in primary health care print a practice that provides safe and effective primary care.

In the present research, the interviewed nurses did not mention the use of the child’s health card as a tool for recording relevant data to the practice of child health surveillance. The child’s health card contains fields for health records of children, and it is fundamental to emphasize the importance of the health team to monitor these data, recording the health information, being prepared to identify problems and harms as early as possible, and performing the active search of children, to contribute to health surveillance^21^.

The articulation among professionals, health services and other social sectors is relevant in the nurses’ performance, also focused on child health surveillance. A study^22^ pointed out that the development of teamwork models and the expansion of nursing practices in primary health care have been recommended by policymakers to meet the population demand. One of the current challenges, though, is how to promote and articulate interprofessional work with integrated and longitudinal management. In the scope of the quality of child health care, a study^23^ expressed the importance of developing a networking model that would bring improvements in child health outcomes through collaborative efforts and adoption of best practices.

In this focus, it is also relevant to highlight the current challenges for advanced nursing practice, a term used to describe a variety of possible nursing functions to exercise an advanced level of practice^24^. Thus, in order to address the health needs in the context of primary health care, nurses work with additional strategies and skills, with knowledge and experience developed within an expanded scope of practice, but one that requires advances and the use of scientific evidence^24^.

Family care and the conditions of home contexts are elements with many contemporary challenges for the attention of health professionals and other sectors that work with families in the communities. In this sense, the nurses’ practices have been fruitful in the face of the precepts of child health surveillance, considering the essential needs of early childhood, situations of vulnerability and adverse and stressful conditions to their development. By doing so, it will be possible to deal with social inequalities, one of the enormous challenges of health care, in order to contribute to systemic social transformations. However, the object of study outlined herein is complex and broad and should be expanded to further research initiatives on health surveillance for the integral care of children in different settings and conditions of health and human development.

This study presents limitations related to the data collected in the interviews without analysis of other secondary data and without the observation of the capillarity of the nursing actions and without a detailed description of interprofessional work.

## Conclusion

In the present study it was possible to appreciate the strategies of FHS nurses used in the provision of care from the point of view of child health surveillance. The focus of such care strategies is in line with the assumptions of child health surveillance aimed at actions that anticipate harm or illnesses and interventions for the promotion, prevention, and continuous follow-up relevant to early childhood development.

The share of nurses in child health surveillance comes in a way that contributes to reducing vulnerabilities because they develop actions with families, favor the capacity of health responses with sharing of knowledge, allow the iprovement of child health indicators and narrow the relationship between health and children’s rights. The needs of children, taking into account the specificities of health conditions, the context of life, human development, the prevention of harm and violence, and diseases prevalent in childhood, the prerogatives of sharing care with families, and articulated work are fundamental and deserve continuous improvement for the transformation of care based on integrality in health.

The work of nurses to provide health care, education and health advocacy, as part of the interprofessional work, may bring advances to the field of community nursing and primary health care, providing a comprehensive range of health promotion actions, disease prevention and interventions for children and their families, towards advanced nursing with a focus on surveillance.

## References

[1] Blair M, Hall D (2006). From health surveillance to health promotion: the changing focus
in preventive children’s services. Arch Dis Child.

[2] Britto PR, Lye SJ, Proelx K, Yousafzai AK, Matthews SG, Vaivada T (2017). Nurturing care: promoting early childhood
development. Lancet.

[3] Dua T, Tomlinson M, Tablante E, Britto P, Yousfzai A, Daelmans B (2016). Global research priorities to accelerate early child development
in the sustainable development era. Lancet.

[4] Black MM, Walker SP, Fernald LCH, Andersen CT, Digirolamo AM, Lu C (2017). Early childhood development coming of age: science through the
life course. Lancet.

[5] Jensen SKG, Bouhouch RR, Walson JL, Daelmans B, Bahl R, Darmstadt GL (2015). Enhancing the child survival agenda to promote, protect, and
support early child development. Semin Perinatol.

[6] Liu L, Oza S, Hogan D, Perin J, Rudan I, Lawn JE (2015). Global, regional and national causes of child mortality in
2000-13, with projections to inform post-2015 priorities: an update
systematic analysis. Lancet.

[7] Almeida PF, Santos AM (2016). Primary Health Care: care coordinator in regionalized
networks. Rev Saúde Pública.

[8] Faria LS, Bertolozzi MR (2010). The surveillance in health basic: perspectives to reach the
surveillance in health. Rev Esc Enferm USP.

[9] Silva TMR, Alvarenga MRM, Oliveira MAC (2012). Evaluation of the vulnerability of families assisted in Primary
Care in Brazil. Rev. Latino-Am. Enfermagem.

[10] Mello DF, Furtado MCC, Fonseca LMM, Pina JC (2012). Child health follow-up and the longitudinality of
caring. Rev Bras Enferm.

[11] Furtado MCC, Braz JC, Pina JC, Mello DF, Lima RAG (2013). Assessing the care of children under one year old in Primary
Health Care. Rev. Latino-Am. Enfermagem.

[12] Olds DL, Kitzman H, Knudtson MD, Anson E, Smith JA, Cole R (2014). Effect of home visiting by nurses on maternal and child
mortality: results of a 2-decade follow-up of a randomized clinical
trial. JAMA Pediatr.

[13] Dmytryshyn AL, Jack SM, Ballantyne M, Wahoush O, Macmillan HL (2015). Long-term home visiting with vulnerable young mothers: an
interpretative description of the impact on public health
nurses. BMC Nurs.

[14] Braun V, Clarke V (2006). Using thematic analysis in psychology. Qual Res Psychol.

[15] Richter LM, Daelmans B, Lombardi J, Heymann J, Boo FL, Behrman JR (2016). Investing in the foundation of sustainable development: pathways
to scale up for early childhood development. Lancet.

[16] Grant J, Lines L, Darbyshire P, Parry Y (2017). How do nurse practitioners work in primary health care settings?
A scoping review.. Int J Nurs Stud.

[17] Appleton JV, Harris M, Oates J, Kelly C (2013). Evaluating health visitor assessments of mother-infant
interactions: a mixed methods study. Int J Nurs Stud.

[18] Monsen KA, Fulkerson JA, Lytton AB, Taft LL, Schwichtenberg LD, Martin KS (2010). Comparing maternal child health problems and outcomes across
public health nursing agencies. Matern Child Health J.

[19] Garuzi M, Achitti MCO, Sato CA, Rocha SA, Spagnuolo RS (2014). User embracement in the Family Health Strategy in Brazil: an
integrative review. Rev Panam Salud Publica.

[20] Swan M, Ferguson S, Chang A, Larson E, Smaldone A (2015). Quality of primary care by advanced practice nurses: a systematic
review. Int J Qual Health Care.

[21] Silva FB, Gaíva MAM, Mello DF (2015). Use of the child health record by families: perceptions of
professionals. Texto Contexto Enferm.

[22] Poghosyan L, Norful AA, Martsolf GR (2017). Primary care nurse practitioner practice characteristics:
barriers and opportunities for interprofessional teamwork. J Ambul Care Manage.

[23] Lannon CM, Peterson LE (2013). Pediatric collaborative improvement networks: background and
overview. Pediatrics.

[24] Oldenburger D, Cassiani SHB, Bryant-Lukosius D, Valaitis RK, Baumann A, Pulcini J (2017). Implementation strategy for advanced practice nursing in primary
health care in Latin America and the Caribbean. Rev Panam Salud Publica.

